# The effects of childhood adversity: Two specific neural patterns

**DOI:** 10.1016/j.neubiorev.2025.106176

**Published:** 2025-04-25

**Authors:** Linlin Yan, Eline J. Kraaijenvanger, Ricardo Wennekers, Veronika I. Müller, Simon B. Eickhoff, Guillén Fernández, Nathalie E. Holz, Nils Kohn

**Affiliations:** a Department of Medical Neuroscience, Donders Institute for Brain, Cognition and Behaviour, Radboud University Medical Center, Nijmegen, the Netherlands; b Department of Child and Adolescent Psychiatry and Psychotherapy, Central Institute of Mental Health, Medical Faculty Mannheim, Heidelberg University, Mannheim, Germany; c German Center for Mental Health (DZPG), partner site Mannheim-Heidelberg-Ulm, Germany; d Institute of Systems Neuroscience, Medical Faculty and University Hospital Düsseldorf, Heinrich Heine University, Düsseldorf, Germany; e Institute of Neuroscience and Medicine, Brain & Behavior (INM-7), Research Centre Jülich, Jülich, Germany

**Keywords:** Childhood adversity, Activation likelihood estimation, Meta-analytic connectivity modeling, Neural network, Functional decoding

## Abstract

Childhood adversity (CA) is associated with an elevated risk of psychopathology across the lifespan and altered brain functions are thought to play an important role in linking CA to mental vulnerability. Previous research has proposed that CA generally influences emotion processing and particularly affects reward processing and cognitive control, yet convergent evidence for CA-related neural and functional networks underlying these processes remains to be fully understood. To investigate the impact of CA on functional brain activations, the present study performed Activation Likelihood Estimation (ALE) analyses across neuroimaging studies involving three task domains: emotion processing, cognitive control, and reward processing. ALE results revealed two significant CA-related convergences of activation in the left amygdala and insula. To better understand and characterize the functions of these ALE-derived clusters, we applied the Meta-Analytic Connectivity Modeling (MACM) approach to identify co-activation maps, and the functional decoding approach to reveal cluster-related psychological processes. Results demonstrated two distinct neural and functional networks in CA: an amygdala-centered emotion processing network and an insula-centered somatomotor processing network. These specific neural patterns indicate the effect of CA on multiple neural and functional networks engaged in sensory-motor and emotion processing functions. Our results provide insights into the neurobiological embedding associated with CA.

## 1. Introduction

Childhood adversity (CA) refers to negative life experiences occurring before reaching adulthood (World Health Organization, 2014). As a broad construct, CA encompasses various forms of child maltreatment such as abuse, neglect, and exposure to violence or war, as well as a set of environmental risk factors such as maternal separation, family poverty, and peer victimization. Consequently, CA can be measured in various ways, either retrospectively or prospectively, through reports from children, their caregivers, or social service records. An epidemiological survey revealed that nearly 40 % of adults worldwide have experienced at least one type of CA (Kessler et al., 2010). Childhood is a crucial stage in overall development, and adverse experiences during this period may result in long-lasting and pervasive consequences for both physical and mental health in adulthood.

Substantial evidence indicates that CA is a strong predictor of the onset of psychopathology across the lifespan (Wade et al., 2022). One central mechanism linking CA to negative mental health outcomes may be the disruption of typical brain development resulting from exposure to adversity. Postnatal adverse experiences during childhood may contribute to either delayed or accelerated brain maturation (Herzberg and Gunnar, 2020; Malave et al., 2022). CA mainly influences brain function in fronto-limbic and dopaminergic circuits, which are implicated in three core cognitive and emotional functions: emotion processing, cognitive control, and reward processing. A heightened or blunted reactivity to emotional stimuli within the amygdala is among the most commonly reported neural alterations in CA studies (Fareri and Tottenham, 2016; Sicorello et al., 2021). Human and animal research has found that individuals who have experienced adversity (e.g., caregiver deprivation or threat exposure) are more likely to exhibit an ’adult-like’ activation pattern in emotional circuits compared to typically developing individuals. This pattern may be reflected in greater interaction between the amygdala and the medial prefrontal cortex (PFC), potentially serving as a more adaptive mechanism for emotion regulation (Callaghan and Tottenham, 2016; Holz et al., 2023). This acceleration of emotion-related neural circuits following CA may come at the expense of relatively slower development in other neural systems (Herzberg and Gunnar, 2020). For instance, the dimensional theory of childhood adversity proposes that specific types of adversity differentially influence neural circuitry, with threat primarily impacting the limbic or fronto-amygdala circuits involved in threat detection and salience processing, whereas deprivation affects the frontoparietal network, potentially delaying cognitive development and executive functioning (McLaughlin et al., 2019).

Empirical evidence on the dimension-specific effects of CA is mixed and inconsistent. Impairment in cognitive control, indicated by decreased recruitment of the PFC, has been found in adolescents exposed not only to early threat but also to deprivation experiences (Leal and Silvers, 2021). Other CA-related neural patterns were also observed in the ventral striatum during reward anticipation and delivery phases, which could reflect the deficits in reward-related functions of responsiveness and approach motivation (Birnie et al., 2020; Novick et al., 2018). Ventral striatal response to rewards also explained the predictive relationship between CA and individual differences in reward-based learning and decision-making behaviors (Kamkar et al., 2017). Overall, differences in brain activation following CA are complex and may also be influenced by multiple factors, including the timing, intensity, and specific types of CA exposure (Holz et al., 2023; Pollok et al., 2022).

Previous CA studies have either focused on the anatomical and functional characteristics within predefined regions of interest (ROIs) using classical paradigms or examined functional connectivity between multiple brain regions during resting or task states. Single experiments are more likely to reveal a wide range of task-based brain activations rather than core regions that are functionally altered following CA exposure. Another challenge is the heterogeneity across neuroimaging studies, including variations in CA measurement, fMRI paradigm design and implementation, and limited sample sizes. Most existing reviews of CA have systematically summarized the general effects of CA on brain function and developed a series of heuristic models to elucidate the neural and psychological mechanisms underlying CA (Cohodes et al., 2021; Smith and Pollak, 2020). However, these qualitative reviews might introduce subjective bias due to the limited statistical power of individual fMRI studies.

Meta-analyses are valuable for further quantifying the neural effects of CA. Coordinate-based Activation Likelihood Estimation (ALE) is a widely used method for identifying convergent regions of neural alterations associated with a specific process or topic (Eickhoff et al., 2012; Turkeltaub et al., 2012). Several meta-analyses published in recent years have also examined the neural effects of CA. For example, an earlier ALE analysis investigated the neural effects of postnatal CA and found BOLD response differences in the right amygdala in both children/adolescents and adults by pooling coordinates from all task domains into a single analysis (Mothersill and Donohoe, 2016). Another study adopted a broader definition of CA—incorporating prenatal factors (e.g., urban environment or toxic exposures) alongside postnatal adversity—and identified two significant clusters: one in the left amygdala related to emotion processing and another in the left precuneus related to memory processing. Pollok et al. (2022) examined the neurostructural traces of CA, revealing age-specific effects on the amygdala and hippocampus, as well as differential effects of various types of maltreatment on the anterior cingulate cortex. Furthermore, a study using Multilevel Kernel Density Analysis (Wager et al., 2007), another meta-analytic approach in neuroimaging, found an association between prior adversity and altered neural activity within the amygdala-prefrontal cortex circuitry across a wide range of task domains (Hosseini-Kamkar et al., 2023). Furthermore, there is still insufficient meta-analytic evidence to delineate the large-scale neural networks influenced by CA. Specifically, how CA-related brain regions coordinate with other areas and the psychological functions they support remain unclear. Meta-Analytic Connectivity Modeling (MACM) provides an opportunity to explore inter-regional functional connectivity by identifying brain regions that are consistently co-activated with a given region. MACM provides a complementary perspective by identifying functionally connected regions that may not be consistently activated in a specific task context (as captured by ALE) but are co-activated with ALE-derived ROIs across broader or more diverse cognitive domains. Functional decoding is another useful approach for further understanding the functional associations of the regions identified by ALE in the context of CA from the perspective of underlying psychological processes.

The first aim of the current study is to employ the ALE algorithm to identify convergent regions of activation across neuroimaging studies examining the main effect of postnatal CA on brain function. This builds on a previous ALE meta-analysis (Kraaijenvanger et al., 2020), which broadly investigated the effects of both prenatal and postnatal CA on brain function. To update the analysis, we will search for and incorporate relevant publications from the past four years into our ALE framework. Given the widespread impact of CA on neural functions and the heterogeneity of brain activation patterns across different task domains (Leal and Silvers, 2021; Malave et al., 2022), we focused on three task domains in this study: cognitive control, emotion processing, and reward processing. A novel perspective suggests that emotion and reward processing are intertwined, potentially sharing overlapping but distinct neural substrates. (Sander and Nummenmaa, 2021). Importantly, similar relationships were also observed in CA and brain activities in both emotion and reward domains (Tottenham, 2020). In this context, a combined affective domain of emotion and reward could be investigated by extracting the coordinates from emotion and reward studies and then comparing the ALE results between combined and separate domain. The second aim is to delineate the co-activating brain regions associated with CA at the neural network level using MACM and to characterize the psychological processes supported by these ALE-derived regions through functional decoding.

## 2. Material and methods

### 2.1. Search strategy

**Fig. 1.**
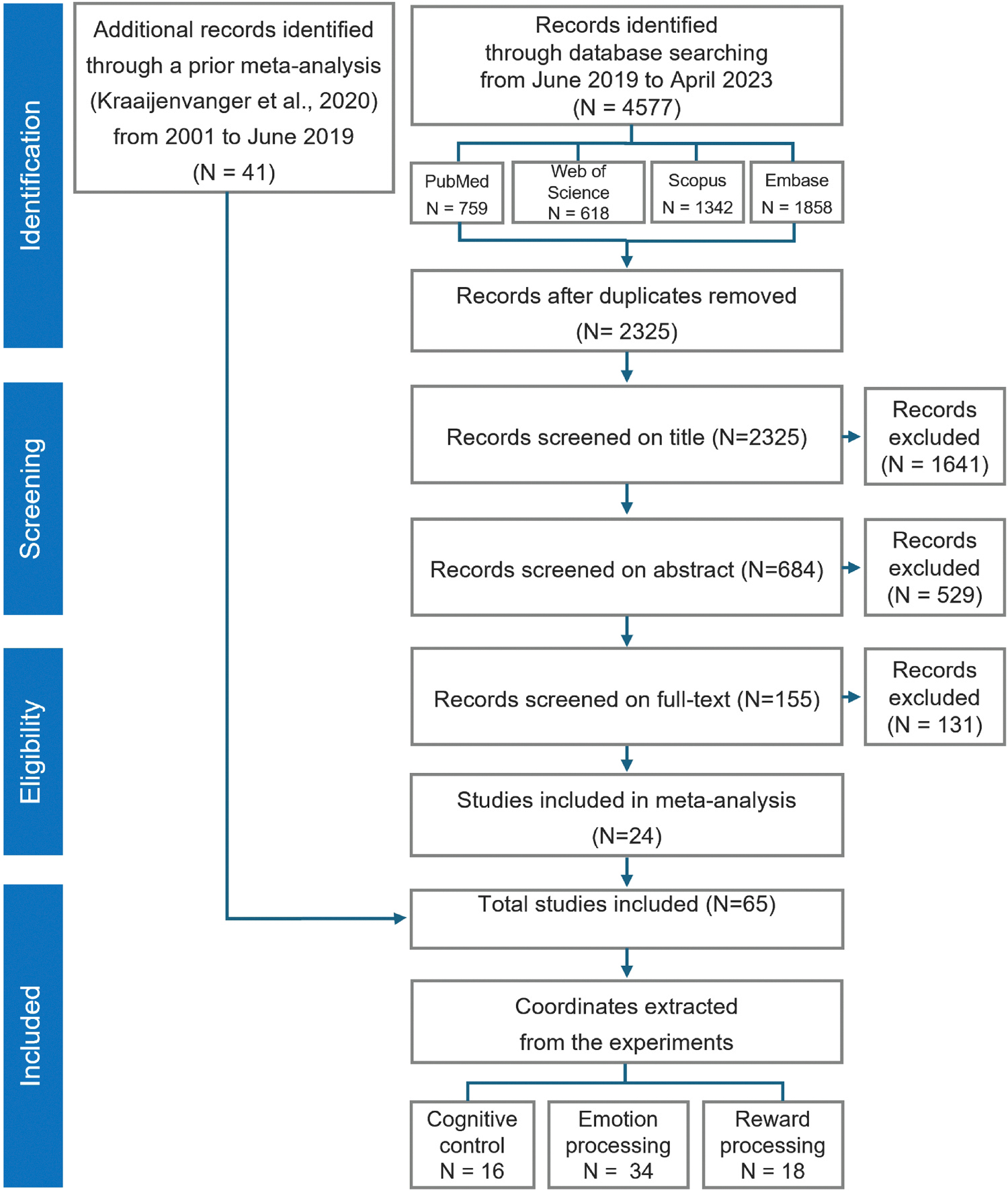
Flowchart outlining the study selection process.

This meta-analysis aims to explore the impact of postnatal CA on the human brain consistently reported in the literature including articles published between 2001 and April 2023. Studies published from 2001 to June 2019 (N = 41) were based on a prior meta-analysis of early-life adversity and human brain functioning (Kraaijenvanger et al., 2020). Given that the current study focuses on postnatal CA, we only included a subset of studies that measured CA after birth and added to this body of studies publications between June 2019 and April 2023 identified by a literature search using four databases: PubMed, Web of Science, Scopus, and Embase (via Ovid). Search strategies were aligned with this previous study (Kraaijenvanger et al., 2020) and generated by encompassing search strings of postnatal adversity and neuroimaging for each database (see Supplementary Table S1). Studies were selected if they: 1) were empirical articles published in English as full-text, 2) included human subjects, 3) completed the peer-review process, 4) measured postnatal adversity before 18 years old using retrospective or prospective methods, and 5) report brain activity differences measured by functional MRI tasks in one or more of three task domains (cognitive control, reward processing, and emotion processing). All full-text articles corresponding to the included abstracts were reviewed by two reviewers (LY and RW) to determine their eligibility based on the inclusion criteria. In cases of disagreement or uncertainty, a senior reviewer (NK) made the final decision. See Fig. 1 for an overview of the study selection process.

### 2.2. Inclusion criteria

Studies were eligible for inclusion if they met the following criteria: 1) performed analyses based on whole-brain coverage rather than using ROIs, 2) reported peak activation coordinates in a standard reference space (MNI or Talairach), and 3) task-based activation maps that included difference coordinates reflecting the main effect of postnatal adversity. These difference coordinates were extracted either from correlation analyses examining the relationship between adversity levels and brain activation within a single group (e.g., correlation of adversity scores with activation while viewing emotional pictures), or from comparisons of brain activity between groups with different levels of adversity, such as adversity-exposed vs. non-exposed or low-adversity vs. high-adversity groups. We also included coordinates that reflected interaction effects between adversity scores and other variables (e.g., gender) on brain activation. Coordinates derived solely from contrasts between different experimental conditions (e.g., negative vs. neutral faces) were not included.

Corresponding authors were contacted via email to request additional information if they did not report peak coordinates from whole-brain analyses or only reported ROI analysis in their articles. Most responses were limited to ROI analyses, with only one study providing difference coordinates from additional whole-brain analyses, which have been incorporated into the present study (Elton et al., 2023). Finally, 65 studies were included in this meta-analysis across three domains: 16 for cognitive control, 15 for reward processing, and 34 for emotion processing (see Supplementary Table S2 for details). For each analysis, to adjust for the within-group effects and repeated measurements, difference coordinates from multiple relevant contrasts within a single study were pooled into one experiment (Müller et al., 2018). An exception to this approach was made for reward processing, as distinct neural substrates are involved at different temporal stages (Jauhar et al., 2021). Specifically, contrasts for anticipation and delivery in these studies (Birn et al., 2017; Morelli et al., 2021; Yang et al., 2021) were treated as independent experiments. Potential convergence will be further examined by calculating contributions to ensure that the results were not driven by overlapping experiments. To ensure the correctness of the standard space (MNI or TAL) and extracted coordinates, the manually recorded data were double-checked by a second investigator.

### 2.3. Activation likelihood estimation (ALE)

The present meta-analysis was performed using the revised ALE algorithm for coordinate-based meta-analyses on functional imaging results (Eickhoff et al., 2012). First, the activation foci in each study were treated as spatial probability distributions centered at the coordinates. Modeled activation maps for each study were generated by combining the activation probabilities for each voxel (Turkeltaub et al., 2012), and these maps were further used to calculate voxel-wise ALE scores for indicating convergence across all the studies. ALE scores are then compared to a null distribution map, which represents a random spatial association between experiments, yielding a statistical map of *p*-values for identifying regions where convergence is greater than chance. We applied the threshold-free cluster enhancement (TFCE) method with a significance threshold of *p* < 0.05 to improve sensitivity in multiple comparisons (Frahm et al., 2022; Noble et al., 2020). Standard TFCE parameter settings (*minimum height* = 0, *H* = 2, *E* = 0.5) were used, as these are recommended and considered optimal for ALE analyses (Smith and Nichols, 2009). Clusters with at least 10 voxels were reported. Statistical significance was assessed at *p* < 0.05.

Studies that reported coordinates in the Talairach space were converted into MNI coordinates using the Lancaster algorithm (Lancaster et al., 2007). Peak coordinates from different experiments involving the same task domain or combined domains were used as inputs for different meta-analyses.^1^ For the main results, we performed four separate ALE meta-analyses: cognitive control (16 experiments from 16 articles), emotion processing (34 experiments from 34 articles), reward processing (18 experiments from 15 articles, due to contrasts from different temporal stages), as well as the combined domain of emotion and reward processing (52 experiments from 48 articles). It is important to note that a meaningful ALE analysis should be performed based on at least 10–15 experiments and an ALE analysis with sufficient power should include at least 17–20 experiments (Eickhoff et al., 2020). As the number of experiments for cognitive control was below the threshold of 17, it is important to note that smaller sample sizes can lead to results being disproportionately influenced by one or two studies. Therefore, we reported the number of experiments contributing to each cluster in the ALE analysis.

### 2.4. Characterization of derived clusters: co-activations

To characterize co-activation patterns across a broad range of tasks within different task domains and gain a better understanding of how the resulting clusters are embedded on a neural network level across neuroimaging experiments, we ran MACM analyses (Eickhoff et al., 2011). First, we defined separate ROIs to reflect the significant clusters from ALE analysis for each individual or combined task domain. For each ROI, the BrainMap database was then used to filter experiments that report at least one focus of activation in the respective ROI. ALE analyses were then conducted across the foci identified in these experiments, ultimately yielding co-activation maps (regions of significant convergence) for each ROI. Similar to a seed voxel approach in connectivity analysis, meta-analytic connectivity maps indicate voxels that are active in studies in which the cluster of interest is active. Co-activation maps were cluster-level corrected for multiple comparisons using FWE correction (*p* < 0.05) and a cluster-forming threshold of *p* < 0.001.

### 2.5. Characterization of derived clusters: functions

We further aimed to functionally describe the significant clusters of ALE results based on the ‘Behavioral Domain (BD)’ and ‘Paradigm Class (PC)’ meta-data categories included in the BrainMap database (Eickhoff et al., 2011). BDs include five main categories of cognition, action, perception, emotion and interoception. PCs classify the specific task employed (for a complete BrainMap taxonomy, see http://brainmap.org/scribe/). Therefore, the functional preference profile of the derived clusters was determined by using the forward (likelihood ratio values) inference method. Forward inference is defined as the probability of a particular task activating a brain region. Thus, we assessed whether the conditional probability of neural activation given a specific psychological process [P(activation|task)] was higher than activation [P(activation)] at the overall chance across the whole BrainMap database in the respective regions. Significance was assessed using a binomial test (*p* < 0.05), corrected for multiple comparisons using the false discovery rate (FDR) method.

Anatomical labeling of MACM and topic-based meta-analyses was performed using in-built FSL atlases, namely the Harvard-Oxford Cortical Atlas, Harvard-Oxford Subcortical Structural Atlas, Juelich Histological Atlas, and MNI Structural Atlas (https://fsl.fmrib.ox.ac.uk/fsl/fslwiki/Atlases). ALE analyses were conducted using scripts based on the revised ALE algorithm (Eickhoff et al., 2012; Turkeltaub et al., 2012), implemented as in-house MATLAB tools (MATLAB version R2024a). We used the same version as in a previous study (Müller et al., 2024) and code of ALE analysis can be found on the Open Science Framework (https://osf.io/dt3kj/?view_only=995297bb53574583b1a0dda978f7f341). MACM analyses were performed using in-house MATLAB tools (MATLAB version R2016b), with scripts implementing the MACM algorithm as described previously (Robinson et al., 2010). Results of ALE and MACM were visualized by Nilearn (https://nilearn.github.io/) and Matplotlib (https://matplotlib.org) using Anaconda (https://www.anaconda.com/) with the virtual environment of Python 3.10. Visualization of functional fingerprints across different ROIs was performed using Python 3.6.8 with the graph library Plotly 5.18.0.

## 3. Results

### 3.1. Meta-analysis: adversity effects on neural activation

**Fig. 2.**
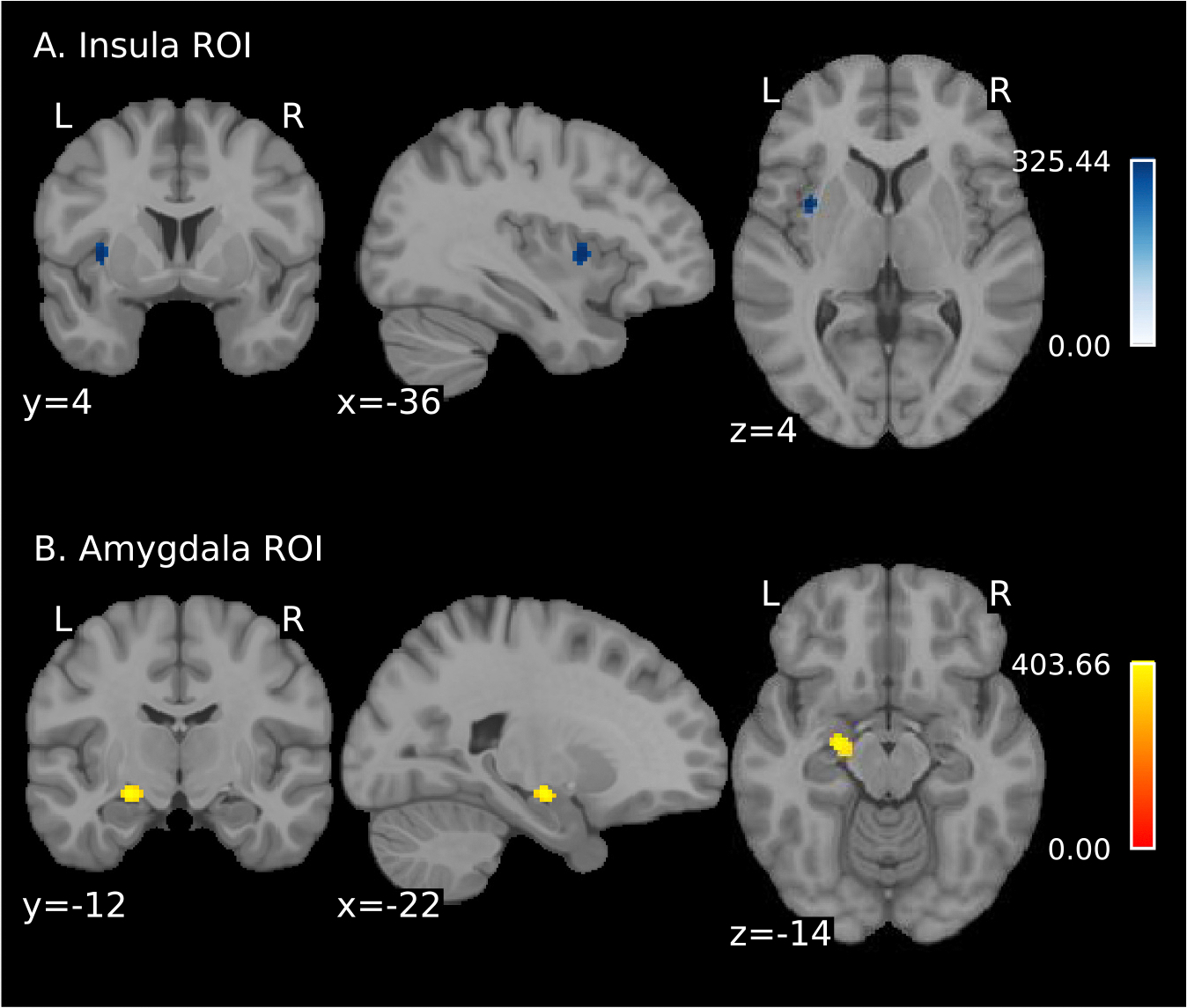
Significant ALE meta-analysis results of the main adversity effect on neural activation across A) cognitive control (blue) and B) emotion processing (yellow). Statistical significance was assessed at *p* < 0.05, TFCE-corrected for multiple comparisons. Colors are coded with TFCE scores. Statistical significance was assessed at *p* < 0.05 (cluster-level FWE corrected for multiple comparisons, cluster-forming threshold *p* < 0.001 at voxel level).

ALE analyses revealed two clusters of significant convergence by examining the main effect of CA on neural activation involved in two different task domains (compare Table 1 and Fig. 2). The ALE analysis for cognitive control included 16 experiments (138 foci; 1022 subjects) and showed one significant cluster of convergence within the left anterior insula (peak MNI [−36, 4, 4], 25 voxels), which we will refer to as Insula ROI. ALE analysis for emotion processing included 34 experiments (380 foci; 2823 subjects) and revealed one significant convergence located in left amygdala and hippocampus (peak MNI [−22,−12,−14], 48 voxels), which we will refer to as Amygdala ROI. The ALE analysis did not indicate any significant convergence of activations for the pool of reward processing (18 experiments, 142 foci; 1523 subjects), as well as the combined pool of emotion and reward processing (52 experiments, 522 foci, 4346 subjects).

### 3.2. Coactivation maps

**Fig. 3.**
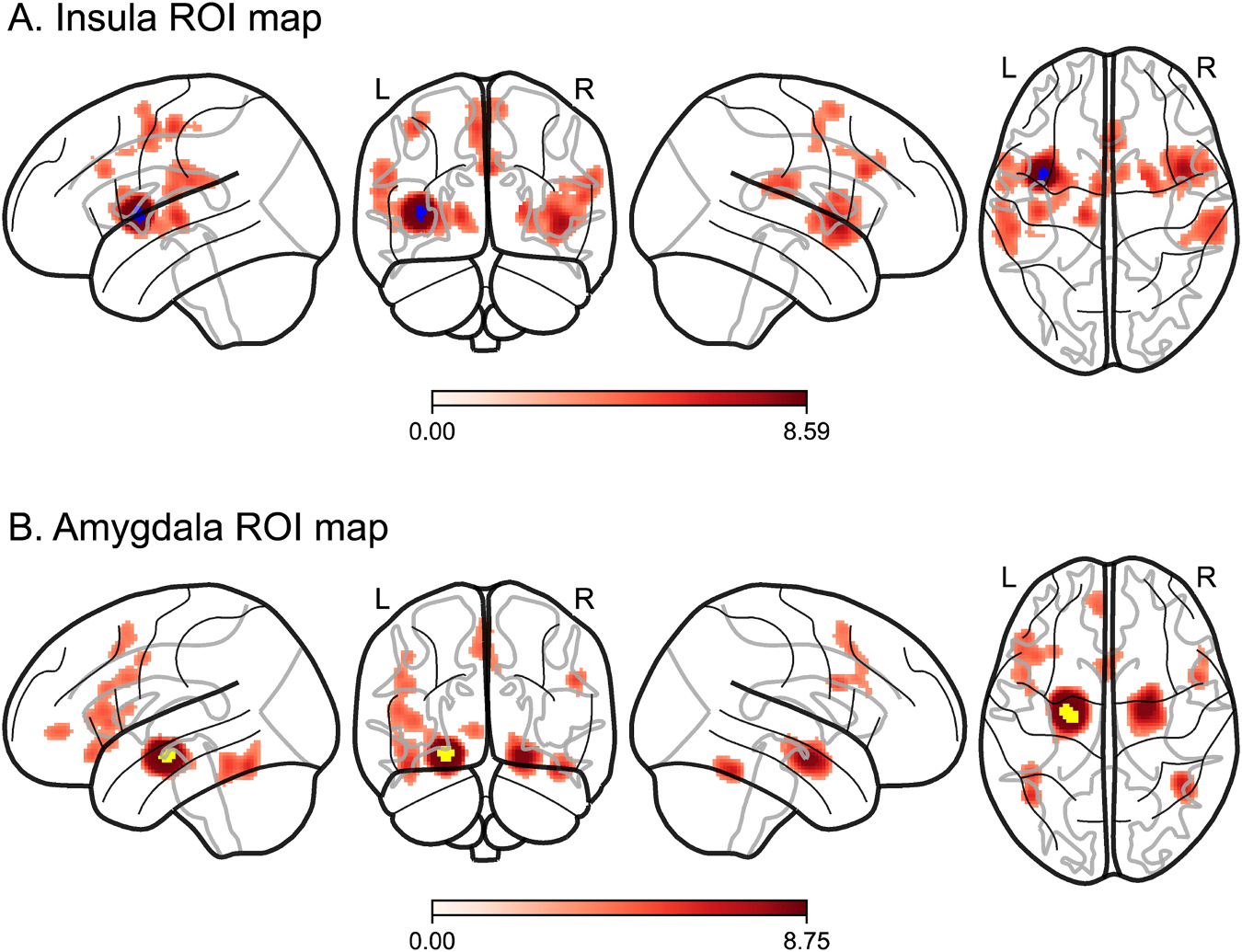
Co-activation maps from MACM using two ROIs derived from ALE meta-analysis. Panel A indicates the co-activation map for the Insula ROI, and Panel B for the Amygdala ROI. Co-activation maps are color-coded in red based on ALE values. ROIs are displayed in blue for cognitive control and yellow for emotion processing.

As shown in Fig 3, MACM analysis of the Insula ROI showed convergent co-activations in bilateral insula, right putamen, precentral and postcentral gyrus, inferior frontal gyrus, secondary somatosensory cortex/parietal operculum (OP1, OP2, and OP4), left thalamus, anterior supramarginal gyrus, inferior parietal lobule, central opercular cortex, anterior cingulate gyrus, supplementary motor cortex, left premotor cortex (BA6), superior frontal gyrus, paracingulate gyrus, primary motor cortex (BA4a) and primary somatosensory cortex (BA1, BA2, BA3b). 61 experiments from the Brain Map database, involving 935 subjects, reported activation within the Insula ROI, resulting in the inclusion of 915 foci across the entire brain in the analysis of the insula ROI.

For the Amygdala ROI, co-activation maps included temporal occipital fusiform cortex, inferior temporal gyrus, occipital fusiform gyrus, bilateral amygdala, bilateral hippocampus, medial geniculate body, frontal orbital and operculum cortex, inferior frontal gyrus, middle frontal gyrus, precentral gyrus, left insula, anterior cingulate gyrus, paracingulate gyrus, supplementary motor cortex, premotor cortex (BA6) and superior frontal gyrus (1312 foci from 100 experiments, 1458 subjects).

### 3.3. Functional characterization

**Fig. 4.**
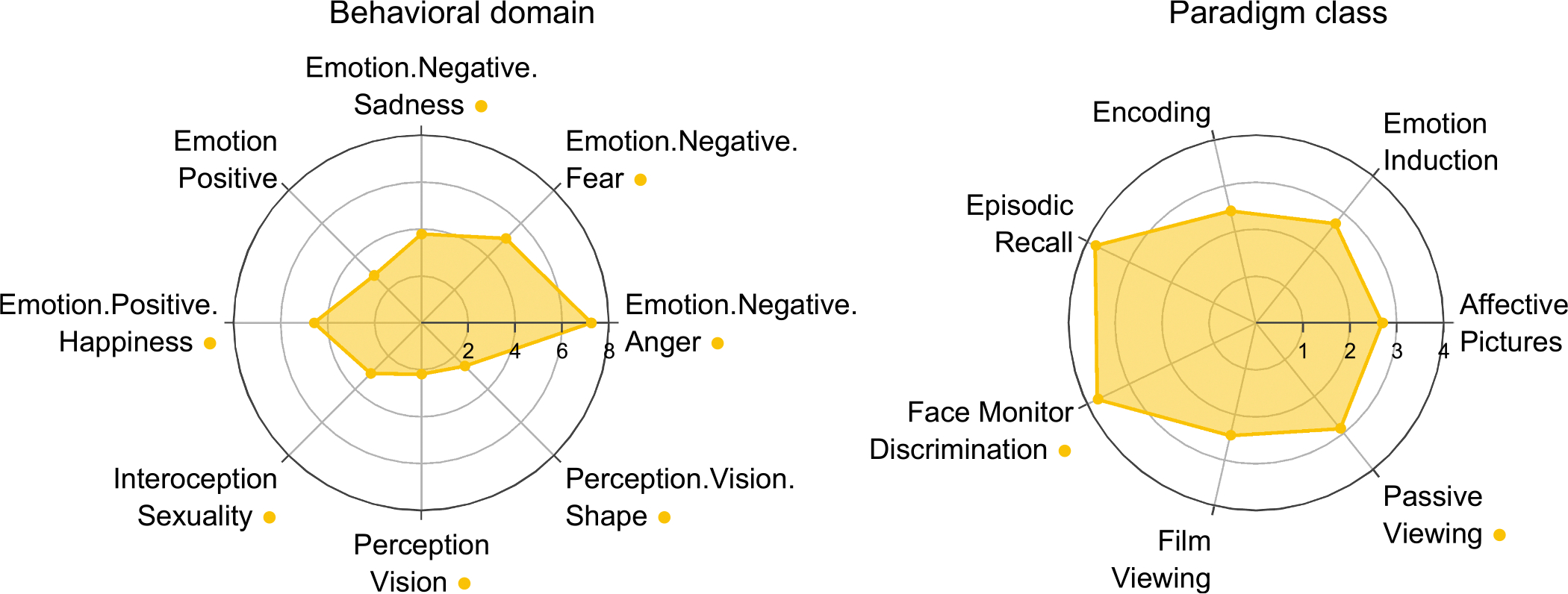
Functional fingerprints of the Amygdala ROI derived from ALE analyses of emotion processing. Statistical significance for each behavioral domain and paradigm class was set at an uncorrected *p* < 0.05, with significance after multiple comparisons indicated by yellow-coded dots next to the name of each label (*p* < 0.05, FDR corrected). Axis labeling indicates likelihood ratio values for forward inference.

Functional characterization according to the BrainMap meta-data was performed for the two derived clusters. The Insula ROI is significantly associated with BDs related to the somesthesis-pain subcategory of perception (FDR-corrected *p* < 0.05) and the execution subcategory of action (uncorrected *p* < 0.05), as well as tasks involving pain monitoring/discrimination for PC (FDR-corrected *p* < 0.05). The Amygdala ROI is associated with BDs related to emotion, perception, and interoception, as well as PCs in emotion induction, face monitoring/discrimination, affective pictures, recall and encoding and film/passive film viewing (for uncorrected and FDR-corrected results, see Fig. 4).

## 4. Discussion

The present study employed the ALE approach to summarize CA-related spatial convergent regions across neuroimaging studies in three task domains that have previously been frequently associated with CA: cognitive control, emotion processing, and reward processing. Consistent with the results of the prior meta-analysis (Kraaijenvanger et al., 2020), the current study identified a cluster in the left amygdala for the emotion processing domain. This finding contrasts with two other studies that pooled all task domains and reported CA effects in the right amygdala (Hosseini-Kamkar et al., 2023; Mothersill and Donohoe, 2016). These discrepancies could suggest that the left amygdala may be specifically involved in emotion processing, whereas the inclusion of heterogeneous task domains in other studies may have obscured this effect. Conversely, the right amygdala effect may be more general but weaker, only emerging in analyses with greater statistical power. Furthermore, our ALE analysis revealed another cluster in the insula, specifically for the cognitive control domain. No significant convergence of activations was found for reward processing, as well as the combined domain of emotion and reward processing. To understand the functions of these CA-related clusters, we performed meta-analytic connectivity analyses and extracted the functional fingerprints to characterize the neural and functional networks of these clusters. In the following, we will integrate the findings from the connectivity analyses and decoding with the functional nature of CA-associated neural activities.

The amygdala is convergently associated with CA in studies from the emotion processing domain. It is a central hub for both negative and positive emotion processing. It has been iconically labelled as ‘the organ of fear’, as well as an important neural contributor to reward learning and decision making (LeDoux, 2003; Wassum, 2022). Co-activations of CA-related amygdala clusters from the emotional domain in this study were observed in the bilateral amygdala and hippocampus, premotor and supplementary motor cortex, anterior cingulate gyrus, paracingulate gyrus, and frontal cortical regions (e.g., inferior frontal gyrus, inferior temporal gyrus, middle frontal gyrus, and superior frontal gyrus). These regions together constitute an emotion processing network centered on the amygdala (Morawetz et al., 2022; Smith and Lane, 2015). Functional characterization underscores an earlier relevance of the amygdala as associated with psychological concepts of perception, memory, and emotion. Our results support the notion that amygdala is an essential gateway to emotions, such that the amygdala is responsible for quickly evaluating sensory inputs (e.g., visual and olfactory) on the basis of multiple factors such as salience, novelty, concern relevance and motivational state, in order to induce an emotional arousal for decoding the significance of the current stimulus (Pignatelli and Beyeler, 2019; Smith and Lane, 2015).

Anatomically, amygdala is a complex subcortical structure located in the anterior medial temporal lobe and has extensive connections with cortical-subcortical areas of the brain, which contribute to the integration with multiple functional systems and modulate a set of adaptive and socio-affective behaviors (Gothard, 2020; Zhang et al., 2021). As such, the amygdala is proposed as a crucial cognitive-emotional connector hub. The amygdala has been proven to engage in a preliminary value integration system by synthesizing concurrent salience signals across cognitive and emotional domains in a decision-making task (Ho et al., 2012; Pessoa, 2017). We also observed the involvement of bilateral hippocampal regions in the coactivation maps of the amygdala, which is consistent with the findings from a meta-analysis of CA studies showing altered functional connectivity between the amygdala and hippocampus (Kraaijenvanger et al., 2023). The neural associations of the hippocampus, as well as its functional labels in memory paradigms (e.g., recall and encoding), might support the strong interplay between emotion and memory, mediated by the amygdala and hippocampus. For instance, high-frequency activities in amygdala and hippocampus both enhance the encoding of emotional memory (Qasim et al., 2023). As discussed in the introduction, consistent evidence indicates that CA is associated with abnormal responses to emotional stimuli (McLaughlin et al., 2019), as well as more difficulties in emotion regulation (Miu et al., 2022), although findings remain mixed and the direction of adversity effect can vary across different dimensions or types of CA. By applying quantitative analysis across neuroimaging studies, the current study underscores the involvement of the amygdala and relevant large-scale emotional network, providing evidence illustrating the central role of the amygdala in the stress response and adaptation system (Zhang et al., 2021).

The area associated with CA in the cognitive control domain was at the border of anterior and posterior insula, which is challenging to characterise, yet has been thought to be primarily responsible for sensorimotor, pain, socioemotional processing, and a set of complex cognitive functions (Uddin et al., 2017). Our meta-analytic connectivity results from the BrainMap database revealed that the insula cluster is functionally connected with frontal and temporal brain regions involved in the somatomotor network, including the inferior frontal gyrus, superior frontal gyrus, anterior and posterior cingulate gyrus, precentral and postcentral gyrus, planum temporale, putamen, and thalamus (Heckner et al., 2021). Co-activation maps of insula cluster indicated the inter-regional neural connections between the insula and the frontal, temporal, and cingulate cortex underscore the effect of CA on the integration of sensory-motor and executive functions. Functional characterizations of the insula cluster were observed in the somesthesis-pain sub-domain of perception, which could be related to incorporating sensory processes into emotional processes. Specifically, the activation of each sensory modality is associated with emotions by recruiting large-scale brain networks of emotion generation and regulation and sensation could also regulate emotions through pathways of modulating attention, reframing negative experiences, and connecting with memory (Boddice and Smith, 2020; Rodriguez and Kross, 2023).

Furthermore, functional association of insula cluster was also found in the execution domain of action. There is also a small overlap in the anterior insula with the connectivity map of amygdala cluster, which might simply be related to the ‘task-active’ nature of this region (Nelson et al., 2010). The relationship between CA severity and adulthood executive dysfunction could be explained by the connectivity strength between sensory-motor networks or the cognitive control network, reflecting the importance of integrating low-level sensory-motor and high-level cognitive processes to achieve optimal executive functions (Silveira et al., 2020). Briefly,the neural pattern of convergent activity showed in the insula cluster and meta-analytic connectivity might provide evidence for the influence of CA on the sensory neural system, indicating that CA is associated with long-lasting patterns of aberrant sensory processing of visual, social, tactile, pain, and olfactory signals (Maier, 2023; Serafini et al., 2016; Tomoda et al., 2009; You and Meagher, 2016). For instance, maltreated individuals might develop an ‘avoidance’ mechanism to limit negative perceptual input into downstream processors, as indicated by dysfunctions in the insula (Mirman et al., 2021). On the other hand, CA-related structural deficits in the primary somatosensory cortex and insula appear to represent neuroplastic adaptations as a consequence of early adverse experiences, promoting avoidance and diminishing approach responses toward trauma (Maier et al., 2020).

Several limitations should be acknowledged in the present meta-analysis. First, the ALE analysis for reward processing did not reveal any significant clusters. One possible explanation could be the high heterogeneity in the paradigms used in reward studies. Future meta-analytic studies could aim to include more reward experiments and classify different reward paradigm classes, for example, based on different stages in the time course of how rewarding stimuli are processed, like reward anticipation or consumption (Oldham et al., 2018). Second, the number of included experiments for cognitive control was 16, which may not be sufficient for performing a well-powered coordinated-based meta-analysis (Eickhoff et al., 2020). Existing systematic reviews have summarized the specific and interactive effects of multiple CA factors to illustrate the association between CA and altered brain functions, such as the type or dimension of adversity, the measurements of CA, and the timing of exposure (McLaughlin et al., 2019; Pollok et al., 2022). Meanwhile, our joint ALE analyses of contrast, correlation, as well as main and interaction effects, might introduce interpretational challenges due to the slightly different nature of contrasts and resultant determination of their conjunction, as well as impact of sample sizes on weighting. However, separate ALE analyses are currently not feasible due to the limited number of available studies. A refined meta-analysis, including more eligible CA studies and comprehensive classification standards, could help further dissect convergent evidence for different sub-patterns of adversity effects and how CA interacts with other factors (e.g., age) to influence brain function.

In summary, our findings provide evidence that postnatal CA is associated with functional alterations in brain regions involved in the processes of emotion processing and cognitive control. These results may enhance our understanding of the neural correlates of CA and how individual differences in brain function are influenced or shaped by early adversity. Importantly, these aberrant neural patterns may serve as mediating pathways linking CA to subsequent negative behavioral, psychological, and biological outcomes. For instance, meta-analyses and reviews indicate that alterations in multiple dimensions of emotion regulation are essential markers of CA, contributing to increased risks of psychopathology and inflammation (Mathur et al., 2022; Miu et al., 2022). Furthermore, previous studies have demonstrated common correlates between adverse childhood experiences and altered cognitive functions (Lund et al., 2022; Wade et al., 2022). Our results extend prior findings of CA effects from higher-order cognitive to basic sensory and somatomotor functions, underscoring the significance of CA on lower-order cognitive functions, which may provide a foundation for higher-order cognition and emotional processes. Within the context of CA, potential interventions aimed at reducing emotional vulnerability or developing adaptive coping strategies, such as cognitive reappraisal, could protect individuals from the negative effects of CA while fostering resilience (Polizzi and Lynn, 2021; Silvers, 2022; Yan and Wu, 2024). Meanwhile, a multidimensional approach may be effective for resilience programs related to CA by combining training targets in both basic and high-level cognitive and emotional processes in the consideration of wide-ranging impacts of CA on distinct and interactive functions.

## 5. Conclusion

The current study investigated the impact of childhood adversity (CA) on brain functional alterations. Our ALE analysis revealed CA-related convergence of activations in the left amygdala and insula. In studies focussing on emotion processing, CA is consistently associated with aberrant activity in an amygdala-centered emotion processing network. In studies focused on cognitive control, an insula-centered somatomotor processing network was associated with CA. These two specific neural patterns support the hypothesis that CA might impact core hubs of separate functional networks, which might relate to the multi-dimensional effects of CA on brain and behaviour (McLaughlin et al., 2019; Smith and Pollak, 2020).

## Supplementary Material

supp

## Figures and Tables

**Table 1:** Clusters showing convergent activation maxima in the ALE meta-analysis.

ALE analyses	Volume (mm^3^)	MNI coordinates			TFCE score	Neuro-anatomical labels	Contributions
		X	Y	Z			

Cognitive control	25	−36	4	4	325.44	Left insula (B44)	4 of 16 experiments (Cará et al., 2019; Lee et al., 2017; Puetz et al., 2016; Thomaes et al., 2012)
Emotion processing	48	−22	−12	−14	402.51	Left amygdala and hippocampus	8 of 34 experiments (De Bellis and Hooper, 2012; Ganzel et al., 2013; Holz et al., 2017; Marusak et al., 2015; McLaughlin et al., 2015; Puetz et al., 2020; Suzuki et al., 2014; Taylor et al., 2006)

## Data Availability

Data will be made available on request.

## Footnotes

1 To explore the effects of distinct types of childhood adversity on brain activation and whether distinct patterns exist across different populations, we first calculated an overall analysis including coordinates from all three task domains. We then conducted additional ALE analyses by dividing this dataset into several independent pools and calculated the following analyses: postnatal adversity (combining all three task domains), children (age < 18), adults (age ≥ 18), disease status (presence of any reported disorder), healthy status (no reported disorder), maltreatment (e.g., abuse and neglect), and specific measurements (i.e., Childhood Trauma Questionnaire scores). Results did not reveal any significant convergence of activations.

## Appendix A. Supporting information

Supplementary data associated with this article can be found in the online version at doi:10.1016/j.neubiorev.2025.106176.
