# Supplementary material for: The effects of childhood adversity: Two specific neural patterns
Source: Neurosci Biobehav Rev. Author manuscript; Available in PMC 2026 Jun 15. (PMC13266811; doi:10.1016/j.neubiorev.2025.106176)
Supplement: supp [file NIHMS2178708-supplement-supp.docx]

**Supplementary Material**

**Table S1.** Search strategies.

**Table S2.** Studies included in the ALE meta-analysis.

**Table S3.** Abbreviations in Table S2.

**References**

**Table S1**. Search strategies.

| PubMed |  |
| --- | --- |
| Search string | (“early stress” OR “early adversit*” OR “early experience*” OR “early environment*” OR “child stress” OR “child maltreatment” OR “child mistreatment” OR “child abuse” OR “child neglect” OR “adverse childhood experience*” OR “adverse childhood event*” OR “prenatal stress” OR “prenatal depression” OR “prenatal anxiety” OR “prenatal exposure” OR “prenatal adversity” OR “childhood socio-economic status” OR “premature birth*” OR “preterm birth*” OR “caregiving quality” OR “caregiving environment” OR “maternal separation” OR “child abuse”[MeSH Terms] OR “adverse childhood experiences”[MeSH Terms] OR “Adult Survivors of Child Adverse Events”[MeSH Terms] OR “Prenatal Exposure Delayed Effects”[MeSH Terms] OR “maternal exposure”[MeSH Terms] OR “maternal deprivation”[MeSH Terms] OR “institutionalization”[MeSH Terms] OR “child poverty”[MeSH Terms] OR “premature birth”[MeSH Terms] OR “infant, low birth weight”[MeSH Terms]) AND (“fMRI” OR “magnetic resonance imaging” OR “functional mri” OR “functional magnetic resonance imaging” OR “mri scan*” OR “magnetic resonance imaging”[MeSH Terms] OR “Positron-Emission Tomography” OR “Positron-Emission Tomography”[MeSH Terms] OR “positron emission tomography imaging” OR “PET” OR “PET scan*” OR “PET imaging”) |
| Initial search (21-04-2023) | 759 articles, with limitations ‘from 2019/06/01 to 2023/04/21 (now)’ AND ‘Humans’ AND ‘English’ |
| Web of Science |  |
| Search string | TS=(“early* stress” OR “early* adversit*” OR “early* experience*” OR “early* environment*” OR “child* stress*” OR “child* maltreatment” OR “child* mistreatment” OR “child* abuse” OR “child* neglect” OR “adverse childhood experience*” OR “adverse childhood event*” OR “prenatal* stress” OR “prenatal* depression” OR “prenatal* anxiety” OR “prenatal* exposure” OR “prenatal* adversity” OR “child* socio-economic status” OR “premature birth*” OR “preterm birth*”OR “caregiving quality” OR “caregiving environment*” OR “maternal separation” OR “maternal deprivation” OR “institutionalization” OR “child* poverty” OR “low birth weight”) AND TS=(“fMRI” OR “magnetic resonance imaging” OR “functional mri” OR “functional magnetic resonance imaging” OR “mri scan*” OR “Positron-Emission Tomography” OR “positron emission tomography imaging” OR “PET” OR “PET scan” OR “PET imaging”) |
| Initial search (21-04-2023) | 618 articles, with limitations ‘from 2019/06/01 to 2023/04/21 (now)’ AND ‘article’ AND ‘English’ |
| Scopus |  |
| Search string | TITLE-ABS-KEY (“early* stress” OR “early* adversit*” OR “early* experience*” OR “early* environment*” OR “child* stress*” OR “child* maltreatment” OR “child* mistreatment” OR “child* abuse” OR “child* neglect” OR “adverse childhood experience*” OR “adverse childhood event*” OR “prenatal* stress” OR “prenatal* depression” OR “prenatal* anxiety” OR “prenatal* exposure” OR “prenatal* adversity” OR “child* socio-economic status” OR “premature birth*” OR “preterm birth*” OR “caregiving quality” OR “caregiving environment*” OR “maternal separation” OR “maternal deprivation” OR “institutionalization” OR “child* poverty” OR “low birth weight”) AND TITLE-ABS-KEY (“fMRI” OR “magnetic resonance imaging” OR “functional mri” OR “functional magnetic resonance imaging” OR “mri scan*” OR “Positron-Emission Tomography” OR “positron emission tomography imaging” OR “PET” OR “PET scan” OR “PET imaging”) |
| Initial search (27-02-2023) | 1342 articles, with limitations ‘2019-now’ AND ‘article’ AND ‘‘human’ OR ‘humans’’ AND ‘English’ |
| Embase  (via Ovid) |  |
| Search string | ('early* stress' OR 'early* adversit*' OR 'early* experience*' OR 'early* environment*' OR 'child* stress*' OR 'child* maltreatment' OR 'child* mistreatment' OR 'child* abuse' OR 'child* neglect' OR 'adverse childhood experience*' OR 'adverse childhood event*' OR 'prenatal* stress' OR 'prenatal* depression' OR 'prenatal* anxiety' OR 'prenatal* exposure' OR 'prenatal* adversity' OR 'child* socio*economic status' OR 'premature birth*' OR 'preterm birth*' OR 'caregiving quality' OR 'caregiving environment*' OR 'maternal separation' OR 'maternal deprivation' OR 'institutionalization' OR 'child* poverty' OR 'low birth weight') AND ('fMRI' OR 'magnetic resonance imaging' OR 'functional mri' OR 'functional magnetic resonance imaging' OR 'mri scan*' OR 'nuclear magnetic resonance imaging' OR 'Positron-Emission Tomography' OR 'positron emission tomography imaging' OR 'PET' OR 'PET scan' OR 'PET imaging') |
| Initial search (27-02-2023) | 1858 articles, Map Term to Subject Heading, with limitations ‘2019-current’ AND ‘Article’ AND ‘Human’ AND ‘English Language’ |

**Table S2**. Studies included in the ALE meta-analysis.

| Article | N | Age Mean | Age SD/Range | Sex (%Male) | Childhood adversity | Task | Contrast | System | # of foci | Software |
| --- | --- | --- | --- | --- | --- | --- | --- | --- | --- | --- |
| Cognitive control |  |  |  |  |  |  |  |  |  |  |
| (Banihashemi et al., 2015) | 135 | 40.7 | 6.16 | 50.3 | Physical abuse (CTQ) | Stroop task/MSIT | incongruent vs congruent | MNI | 10 | SPM8 |
| (Blair et al., 2019) | 116 | 15 | 10~18 | 60.3 | Childhood adversity (CTQ) | Affective Stroop task | incongruent/congruent vs. View | TAL | 13 | AFNI |
| (Bruce et al., 2013) | 22 | 10.9 | 9~12 | 54.5 | Foster care / maltreatment (CPS report) | Go/NoGo task | correct NoGo-correct Go incorrect NoGo-correct NoGo | MNI | 4 3 | SPM8 |
| (Cará et al., 2019) | 37 | 11.43 | 9~14 | 67.6 | Violence exposure (JVQ-R2) | Change task | change vs. go | MNI | 22 | AFNI |
| (Demers et al., 2022) | 72 | 30.18 | unknown | 51.4 | Childhood maltreatment (Documented records/Maternal Maltreatment Classification Interview) | IAPS Go/No-Go task | negative > neutral | MNI | 3 | FSL |
| (Elton et al., 2023) | 144 | unknown | 18~19 | 34 | Childhood maltreatment (CTQ) | Stop Signal task | successful stop minus go | MNI | 5 | AFNI |
| (Harms et al., 2017) | 33 | 20.6 | 19~23.7 | 45.5 | Early life stress (YLSI) | Go/NoGo task | NoGo error > NoGo correct | MNI | 1 | AFNI |
| (Lee et al., 2017) | 23 | 16.2 | unknown | 100 | Parent or peer verbal abuse (VAQ) | Emotional Stroop task | negative > neutral | MNI | 25 | FSL |
| (Lim et al., 2015) | 66 | 17.3 | 13~20 | 66.7 | Maltreatment (CTQ / CECA) | Stop Signal task | failed stop > successful go | MNI | 4 | SPM8 |
| (Seghete et al., 2017) | 32 | 26 | 23~30 | 0 | Childhood adversity (THQ) | Emotional Stroop task | positive > negative | MNI | 17 | FSL |
| (Miller et al., 2015) | 98 | 33.6 | 13.1 | 52 | Maltreatment (ELSQ) | Go/NoGo task | NoGo-Go | MNI | 5 | SPM8 |
| (Mueller et al., 2010) | 33 | 13 | unknown | 42.4 | Institutionalization/ maltreatment (KSADS) | Change task | correct change vs correct Go correct change vs incorrect change | MNI | 8 3 | SPM99 |
| (Puetz et al., 2016) | 36 | 12.7 | 10~14 | 45 | Maltreatment (CTQ) | Emotional Stroop task | negative-neutral words negative-incongruent color words | MNI | 12 3 | SPM8 |
| (Thomaes et al., 2012) | 51 | 34.3 | unknown | 0 | Child abuse | Stroop task | incongruent vs congruent | MNI | 9 | SPM5 |
| (van Rooij et al., 2020) | 69 | 10.83 | 8~14 | 47.8 | Trauma exposure (TESI) Violence exposure (VEX-R) | Emotional Go/NoGo task | NoGo>Go | MNI | 4 | SPM8 |
| (Wymbs et al., 2020) | 55 | 11.71 | unknown | 46.2 | Childhood adversity (Y-VACS) | Emotional Go/NoGo task | Fear > Neutral | MNI | 1 | SPM12 |
| Reward processing |  |  |  |  |  |  |  |  |  |  |
| (Birn et al., 2017) | 42 | 20.5 | 19~23.7 | Mixed | Childhood stress exposure (YLSI; semi-structured questions) | Monetary incentive delay task | reward > no win loss > no loss (both incl. anticipation and response) | MNI | 10 2 | AFNI |
| (Armbruster-Genç et al., 2022) | 58 | 13.5 | 10~16 | 37.9 | Physical abuse and/or intimate partner violence (social service) | Effort-based decision-making task | decision: high effort > low effort | MNI | 1 | SPM12 |
| (Blair et al., 2022) | 142 | 16.4 | 1.2 | Mixed | CTQ | Passive avoidance learning task | Reward vs Punishment | TAL | 4 | AFNI |
| (DelDonno et al., 2019) | 50 | 27.28 | 18~55 | 22 | Childhood adversity (CTQ) | Monetary incentive delay task | anticipation: win>neutral | MNI | 12 | SPM8 FSL |
| (Gianaros et al., 2011) | 76 | 44.6 | 31~54 | 46.1 | SES (parental education in 18 years) | Card-guessing game | gain > loss gain > control | MNI | 2 2 | SPM2 |
| (Gonzalez et al., 2016) | 77 | 24.41 | 1.11 | 50.6 | Neighborhood quality (NQQ) | Monetary incentive delay task | anticipation: reward > neutral | MNI | 8 | FSL |
| (Hanson et al., 2016) | 72 | 26.3 | 1.1 | 100 | Stressful life events (LC measure) | Card-guessing game | win > loss | MNI | 4 | AFNI |
| (Holz et al., 2017) | 171 | 25 | unknown | 40.4 | Childhood family adversity (standardized parent interview) | Reward paradigm | anticipation: monetary > verbal | MNI | 55 | SPM8 |
| (Jorgensen et al., 2023) | 165 | 12.89 | 0.56 | Mixed | Neighborhood disadvantage and SES (parents report) | Social incentive delay task | Anticipation: threat > neutral | MNI | 1 | SPM12 |
| (Kim‐Spoon et al., 2019) | 135 | 15.05 | 0.54 | 52 | Family Multi-Risk Index | Lottery choice task | Decision: high risk > low risk | MNI | 16 | SPM8 |
| (Morelli et al., 2021) | 46 | 7.52 | 5.9~9.6 | Mixed | Early life stress (parents report) | Monetary incentive delay task | Anticipation: Reward > No reward Feedback: Reward >No Reward Feedback: Hit > Miss | TAL | 1 8 1 | AFNI |
| (Romens et al., 2015) | 123 | 16.4 | unknown | 0 | Childhood socioeconomic disadvantage (need of public assistance) | Monetary reward guessing task | reward anticipation > baseline | MNI | 5 1 | SPM8 |
| (Seitz et al., 2023) | 118 | 31.1 | 11 | Mixed | ACE (maternal antipathy) | Monetary and social reward task | Anticipation: reward > neutral | MNI | 12 | SPM12 |
| (Takiguchi et al., 2015) | 36 | 12.7 | unknown | 50 | Maltreatment (CATS) | Gambling task | reward > no reward | MNI | 2 | SPM8 |
| (Yang et al., 2021) | 45 | 14.92 | 1.9 | Mixed | CTQ | Reward processing | Anticipation: Reward > No reward Feedback: Hit > Miss | TAL | 20 | AFNI |
| Emotion processing |  |  |  |  |  |  |  |  |  |  |
| (Aas et al., 2017) | 101 | 31.9 | 10.1 | 55 | Maltreatment (CTQ) | Emotional faces task | negative vs positive | MNI | 2 | FSL (v-6) |
| (Blair et al., 2020) | 47 | 15.1 | unknown | 31.9 | Sexual abuse (CTQ) | Looming task | looming vs. receding human vs. animal threatening vs. neutral | TAL | 23 | AFNI |
| (Colich et al., 2017) | 98 | 11.42 | 1.08 | unknown | Early life stress (TESI-C) | Emotional regulation task | label vs match | MNI | 1 | FSL (v-6) |
| (Dannlowski et al., 2013) | 134 | 34.5 | 10.6 | 47.3 | Maltreatment (CTQ) | Subliminal affective priming paradigm | sad > happy | MNI | 9 (12) | SPM8 |
| (De Bellis & Hooper, 2012) | 16 | 13.6 | 11.6~18.1 | 50 | Abuse/Neglect (CPS report) | Emotional oddball task | sad > neutral | MNI | 5 | SPM |
| (Fonzo et al., 2013) | 33 | 39.3 | 8.46 | 0 | Maltreatment (CTQ) | Emotional faces task | angry > shape | TAL | 2 | AFNI |
| (Ganzel et al., 2013) | 14 | 13.1 | 2.2 | 71.4 | Life events (CIDI/parent report) | Emotional faces task | negative > neutral | MNI | 1 | SPM8 |
| (Gee et al., 2013) | 89 | 11.6 | 6.5~17.6 | 51.7 | Maternal deprivation | Emotional faces task | fear > happy | TAL | 2 | AFNI |
| (Gerhardt et al., 2023) | 62 | 39.5 | unknown | 56.5 | CTQ | Face matching task | No vs Habituation | MNI | 1 | SPM12 |
| (Hart et al., 2018) | 67 | 17.3 | 12~20 | 64.2 | Abuse (CTQ/CECA) | Emotion discrimination task | negative > fixation negative > positive | MNI | 3 3 | SPM8 |
| (Herringa et al., 2013) | 28 | 26.6 | 22.2~31.8 | 100 | Maltreatment (CTQ) | Dynamic face task | negative > shape | MNI | 3 | SPM8 |
| (Holz et al., 2017) | 181 | 25 | unknown | 40.4 | Family adversity (parent interview) | Face-matching task | fear/angry face > shape | MNI | 11 | SPM8 |
| (Jedd et al., 2015) | 71 | 30.1 | 23~37 | 47.9 | Maltreatment (CPS report/MMCI) | Emotion-matching task | face > shape | MNI | 9 | FSL (v. 4.1.9) |
| (Jenness et al., 2021) | 151 | 12.63 | 2.68 | 49.7 | Physical or sexual abuse (self-/parent report and interview) | Emotion regulation task | Look-Negative > Look-Neutral | MNI | 8 | FSL |
| (Keding & Herringa, 2016) | 53 | 14.3 | 8.07~18.80 | 32.2 | Childhood trauma (KSADS) | Dynamic face task | negative (angry) > shape positive (happy) > shape | MNI | 1 1 | FSL and AFNI |
| (Kim et al., 2013) | 49 | 23.6 | 20~27 | 55.1 | Childhood poverty (I/N ratio) | Emotion regulation task | reappraise > maintain | MNI | 12 | AFNI |
| (Lang et al., 2012) | 44 | 27.1 | unknown | 0 | Maltreatment (CTQ) | Emotion regulation task | upregulate > maintain downregulate > maintain | MNI | 10 3 | SPM8 |
| (Lee et al., 2015) | 31 | 16.1 | 0.48 | 100 | Verbal abuse (VAQ) | Emotion/gender identification task | negative > neutral | MNI | 13 | FSL |
| (Marusak et al., 2015a) | 33 | 12.3 | unknown | 24.2 | Childhood trauma (CTACTSC) | Emotional conflict task | incongruent-congruent | MNI | 7 | SPM8 |
| (Marusak et al., 2015b) | 30 | 12.73 | unknown | 20 | Childhood trauma (CTACTSC) | Emotional conflict task | incongruent-congruent postincongruent incongruent minus postcongruent incongruent | MNI | 14 14 | SPM8 |
| (McCrory et al., 2013) | 41 | 12.4 | unknown | 56.1 | Maltreatment (case files) | Masked dot-probe paradigm | negative > neutral positive > neutral | MNI | 1 4 | SPM8 |
| (McLaughlin et al., 2015) | 42 | 16.6 | 1.41 | 38.1 | Abuse (CTQ/CECA) Questionnaire and interview | Emotion regulation task | look negative > look neutral  decrease > look neutral | MNI | 8 2 | FSL |
| (Nagy et al., 2021) | 61 | 33 | 18~54 | 65.6 | CTQ | Facial emotion recognition | Shape matching > Face matching | MNI | 6 | FSL |
| (Neukel et al., 2019) | 53 | 39.4 | unknown | 0 | Maltreatment (CECA) | Emotional face recognition task | positive > neutral | MNI | 7 | SPM8 |
| (Nicol et al., 2015) | 36 | 35.4 | 20~53 | 13.9 | Childhood trauma (CTQ) | Emotional faces task | negative > neutral | MNI | 3 |  |
| (Park et al., 2022) | 70 | 8.36 | 1.74 | 69 | SES Child stressful life events (parents report) | Movie-watching (positive and negative emotional events, and rich parent-child interactions) | Watch>Rest | MNI | 3 | FSL |
| (Peters et al., 2019) | 132 | 26.04 | unknown | 31.8 | Childhood adversity (CTQ) | Emotional face matching task | angry > shape fear > shape | MNI | 2 18 | SPM8 |
| (Puetz et al., 2020) | 414 | 19.7 | 18~22 | 39.6 | Childhood adversity (CTQ) | Face-matching task | angry > neutral | MNI | 91 | SPM8 |
| (Quidé et al., 2017) | 109 | 39.8 | unknown | 43.1 | CTQ-SF | Emotional face-matching task | Face matching > Shape matching | MNI | 4 | SPM12 |
| (Suzuki et al., 2014) | 115 | 9.9 | 1.33 | 48.7 | Early life trauma / stress (PAPA/CAPA) | Emotional faces task | negative > neutral positive > neutral | MNI | 73 44 | FIDL analysis package |
| (Taylor et al., 2006) | 30 | unknown | unknown | 40 | Family stress (RFQ) | Emotion/gender identification task | observe > fixation | MNI | 1 | SPM99 |
| (Wainsztein et al., 2021) | 59 | 41.05 | unknown | 33.9 | Childhood adversity (ACE) | Emotion regulation task | regulate > observe | MNI | 21 | SPM12 |
| (Weissman et al., 2020) | 149 | 12.77 | 8~17 | 49.7 | Violence exposure (CECA, VEX-R) Childhood adversity (CTQ, PTSD-RI) Caregiver support (CTS, JVQ, PTSD-RI) | Emotional faces task | fear > scrambled calm > scrambled | MNI | 5 4 | FSL |
| (Weissman et al., 2022) | 177 | 11 | 10~13 | 46.9 | Threat and deprivation experiences  (Parent or child report or interview) | Emotional faces task | Fearful > Neutral | MNI | 1 | FSL and AFNI |

*Notes*. Abbreviations are indicated in Table S3.

**Table S3.** Abbreviations in Table S2.

| Abbreviations | Full name |
| --- | --- |
| ACE | Adverse Childhood Experience |
| CATS | Child Abuse and Trauma Questionnaire |
| CECA | Childhood Experience of Care and Abuse interview |
| CIDI | Composite International Diagnostic Interview |
| CPS | Child protective services |
| CTACTSC | Children’s Trauma Assessment Center Trauma Screening Checklist |
| CTQ | Childhood Trauma Questionnaire |
| CTQ-SF | Childhood Trauma Questionnaire Short-Form |
| ELSQ | Early Life Stress Questionnaire |
| I/N ratio | Income-to-need ratio |
| IAPS | [International Affective Picture System](https://www.umass.edu/research/guidance/international-affective-picture-system-iaps) |
| JVQ-R2 | Juvenile Victimization Questionnaire 2nd revision |
| KSADS | Kiddie Schedule for Affective Disorders and Schizophrenia |
| LC | Life Changes measure |
| MSIT | Multi-source interference task |
| NQQ | Neighborhood Quality Questionnaire |
| PAPA/CAPA | Preschool Age Psychiatric Assessment / Child and Adolescent Psychiatric Assessment |
| PTSD-RI | the UCLA Posttraumatic Stress Disorder Reaction Index |
| RFQ | Risky Families Questionnaire |
| SES | Socioeconomic Status |
| TESI | Traumatic Events Screening Inventory |
| TESI-C | Traumatic Events Screening Inventory for Children |
| THQ | Trauma History Questionnaire |
| VAQ | Verbal Abuse Questionnaire |
| VEX-R | Violence Exposure Scale for Children-Revised |
| YLSI | Youth Life Stress Interview |
| Y-VACS | Yale-Vermont Adversity in Childhood Scale |

**References**

Aas, M., Kauppi, K., Brandt, C., Tesli, M., Kaufmann, T., Steen, N. E., Agartz, I., Westlye, L., Andreassen, O. A., & Melle, I. (2017). Childhood trauma is associated with increased brain responses to emotionally negative as compared with positive faces in patients with psychotic disorders. *Psychological medicine*, *47*(4), 669-679.

Armbruster-Genç, D. J., Valton, V., Neil, L., Vuong, V., Freeman, Z., Packer, K. C., Kiffin, M. J., Roiser, J. P., Viding, E., & McCrory, E. (2022). Altered reward and effort processing in children with maltreatment experience: a potential indicator of mental health vulnerability. *Neuropsychopharmacology*, *47*(5), 1063-1070.

Banihashemi, L., Sheu, L. K., Midei, A. J., & Gianaros, P. J. (2015). Childhood physical abuse predicts stressor-evoked activity within central visceral control regions. *Social Cognitive and Affective Neuroscience*, *10*(4), 474-485.

Birn, R. M., Roeber, B. J., & Pollak, S. D. (2017). Early childhood stress exposure, reward pathways, and adult decision making. *Proceedings of the National Academy of Sciences*, *114*(51), 13549-13554.

Blair, K. S., Aloi, J., Bashford-Largo, J., Zhang, R., Elowsky, J., Lukoff, J., Vogel, S., Carollo, E., Schwartz, A., & Pope, K. (2022). Different forms of childhood maltreatment have different impacts on the neural systems involved in the representation of reinforcement value. *Developmental cognitive neuroscience*, *53*, 101051.

Blair, K. S., Aloi, J., Crum, K., Meffert, H., White, S. F., Taylor, B. K., Leiker, E. K., Thornton, L. C., Tyler, P. M., & Shah, N. (2019). Association of different types of childhood maltreatment with emotional responding and response control among youths. *JAMA network open*, *2*(5), e194604-e194604.

Blair, K. S., Bashford-Largo, J., Shah, N., Lukoff, J., Elowsky, J., Vogel, S., Emmert, A., Zhang, R., Dobbertin, M., & Pollak, S. (2020). Sexual abuse in adolescents is associated with atypically increased responsiveness within regions implicated in self-referential and emotional processing to approaching animate threats. *Frontiers in psychiatry*, *11*, 345.

Bruce, J., Fisher, P. A., Graham, A. M., Moore, W. E., Peake, S. J., & Mannering, A. M. (2013). Patterns of brain activation in foster children and nonmaltreated children during an inhibitory control task. *Development and psychopathology*, *25*(4pt1), 931-941.

Cará, V. M., Esper, N. B., De Azeredo, L. A., Iochpe, V., Dalfovo, N. P., Santos, R. C., Sanvicente-Vieira, B., Grassi-Oliveira, R., Franco, A. R., & Buchweitz, A. (2019). An fMRI study of inhibitory control and the effects of exposure to violence in Latin-American early adolescents: Alterations in frontoparietal activation and performance. *Social Cognitive and Affective Neuroscience*, *14*(10), 1097-1107.

Colich, N. L., Williams, E. S., Ho, T. C., King, L. S., Humphreys, K. L., Price, A. N., Ordaz, S. J., & Gotlib, I. H. (2017). The association between early life stress and prefrontal cortex activation during implicit emotion regulation is moderated by sex in early adolescence. *Development and psychopathology*, *29*(5), 1851-1864.

Dannlowski, U., Kugel, H., Huber, F., Stuhrmann, A., Redlich, R., Grotegerd, D., Dohm, K., Sehlmeyer, C., Konrad, C., & Baune, B. T. (2013). Childhood maltreatment is associated with an automatic negative emotion processing bias in the amygdala. *Human Brain Mapping*, *34*(11), 2899-2909.

De Bellis, M. D., & Hooper, S. R. (2012). Neural substrates for processing task‐irrelevant emotional distracters in maltreated adolescents with depressive disorders: a pilot study. *Journal of Traumatic Stress*, *25*(2), 198-202.

DelDonno, S. R., Mickey, B. J., Pruitt, P. J., Stange, J. P., Hsu, D. T., Weldon, A. L., Zubieta, J.-K., & Langenecker, S. A. (2019). Influence of childhood adversity, approach motivation traits, and depression on individual differences in brain activation during reward anticipation. *Biological Psychology*, *146*, 107709.

Demers, L. A., Hunt, R. H., Cicchetti, D., Cohen-Gilbert, J. E., Rogosch, F. A., Toth, S. L., & Thomas, K. M. (2022). Impact of childhood maltreatment and resilience on behavioral and neural patterns of inhibitory control during emotional distraction. *Development and psychopathology*, *34*(4), 1260-1271.

Elton, A., Allen, J. H., Yorke, M., Khan, F., Xu, P., & Boettiger, C. A. (2023). Sex moderates family history of alcohol use disorder and childhood maltreatment effects on an fMRI stop‐signal task. *Human Brain Mapping*, *44*(6), 2436-2450.

Fonzo, G. A., Flagan, T. M., Sullivan, S., Allard, C. B., Grimes, E. M., Simmons, A. N., Paulus, M. P., & Stein, M. B. (2013). Neural functional and structural correlates of childhood maltreatment in women with intimate-partner violence-related posttraumatic stress disorder. *Psychiatry Research: Neuroimaging*, *211*(2), 93-103.

Ganzel, B. L., Kim, P., Gilmore, H., Tottenham, N., & Temple, E. (2013). Stress and the healthy adolescent brain: Evidence for the neural embedding of life events. *Development and psychopathology*, *25*(4pt1), 879-889.

Gee, D. G., Gabard-Durnam, L. J., Flannery, J., Goff, B., Humphreys, K. L., Telzer, E. H., Hare, T. A., Bookheimer, S. Y., & Tottenham, N. (2013). Early developmental emergence of human amygdala–prefrontal connectivity after maternal deprivation. *Proceedings of the National Academy of Sciences*, *110*(39), 15638-15643.

Gerhardt, S., Berhe, O., Moessnang, C., Horning, M., Kiefer, F., Tost, H., & Vollstädt‐Klein, S. (2023). Lack of amygdala habituation to negative emotional faces in alcohol use disorder and the relation to adverse childhood experiences. *Addiction biology*, *28*(1), e13251.

Gianaros, P. J., Manuck, S. B., Sheu, L. K., Kuan, D. C., Votruba-Drzal, E., Craig, A. E., & Hariri, A. R. (2011). Parental education predicts corticostriatal functionality in adulthood. *Cerebral Cortex*, *21*(4), 896-910.

Gonzalez, M. Z., Allen, J. P., & Coan, J. A. (2016). Lower neighborhood quality in adolescence predicts higher mesolimbic sensitivity to reward anticipation in adulthood. *Developmental cognitive neuroscience*, *22*, 48-57.

Hanson, J. L., Albert, D., Iselin, A.-M. R., Carre, J. M., Dodge, K. A., & Hariri, A. R. (2016). Cumulative stress in childhood is associated with blunted reward-related brain activity in adulthood. *Social Cognitive and Affective Neuroscience*, *11*(3), 405-412.

Harms, M. B., Birn, R., Provencal, N., Wiechmann, T., Binder, E. B., Giakas, S. W., Roeber, B. J., & Pollak, S. D. (2017). Early life stress, FK506 binding protein 5 gene (FKBP5) methylation, and inhibition-related prefrontal function: a prospective longitudinal study. *Development and psychopathology*, *29*(5), 1895-1903.

Hart, H., Lim, L., Mehta, M. A., Simmons, A., Mirza, K., & Rubia, K. (2018). Altered fear processing in adolescents with a history of severe childhood maltreatment: an fMRI study. *Psychological medicine*, *48*(7), 1092-1101.

Herringa, R. J., Phillips, M. L., Fournier, J. C., Kronhaus, D. M., & Germain, A. (2013). Childhood and adult trauma both correlate with dorsal anterior cingulate activation to threat in combat veterans. *Psychological medicine*, *43*(7), 1533-1542.

Holz, N. E., Boecker-Schlier, R., Buchmann, A. F., Blomeyer, D., Jennen-Steinmetz, C., Baumeister, S., Plichta, M. M., Cattrell, A., Schumann, G., & Esser, G. (2017). Ventral striatum and amygdala activity as convergence sites for early adversity and conduct disorder. *Social Cognitive and Affective Neuroscience*, *12*(2), 261-272.

Jedd, K., Hunt, R. H., Cicchetti, D., Hunt, E., Cowell, R. A., Rogosch, F. A., Toth, S. L., & Thomas, K. M. (2015). Long-term consequences of childhood maltreatment: Altered amygdala functional connectivity. *Development and psychopathology*, *27*(4pt2), 1577-1589.

Jenness, J. L., Peverill, M., Miller, A. B., Heleniak, C., Robertson, M. M., Sambrook, K. A., Sheridan, M. A., & McLaughlin, K. A. (2021). Alterations in neural circuits underlying emotion regulation following child maltreatment: A mechanism underlying trauma-related psychopathology. *Psychological medicine*, *51*(11), 1880-1889.

Jorgensen, N. A., Muscatell, K. A., McCormick, E. M., Prinstein, M. J., Lindquist, K. A., & Telzer, E. H. (2023). Neighborhood disadvantage, race/ethnicity and neural sensitivity to social threat and reward among adolescents. *Social Cognitive and Affective Neuroscience*, *18*(1), nsac053.

Keding, T. J., & Herringa, R. J. (2016). Paradoxical prefrontal–amygdala recruitment to angry and happy expressions in pediatric posttraumatic stress disorder. *Neuropsychopharmacology*, *41*(12), 2903-2912.

Kim‐Spoon, J., Lauharatanahirun, N., Peviani, K., Brieant, A., Deater‐Deckard, K., Bickel, W. K., & King‐Casas, B. (2019). Longitudinal pathways linking family risk, neural risk processing, delay discounting, and adolescent substance use. *Journal of child psychology and psychiatry*, *60*(6), 655-664.

Kim, P., Evans, G. W., Angstadt, M., Ho, S. S., Sripada, C. S., Swain, J. E., Liberzon, I., & Phan, K. L. (2013). Effects of childhood poverty and chronic stress on emotion regulatory brain function in adulthood. *Proceedings of the National Academy of Sciences*, *110*(46), 18442-18447.

Lang, S., Kotchoubey, B., Frick, C., Spitzer, C., Grabe, H. J., & Barnow, S. (2012). Cognitive reappraisal in trauma-exposed women with borderline personality disorder. *Neuroimage*, *59*(2), 1727-1734.

Lee, S. W., Choi, J., Lee, J.-S., Yoo, J. H., Kim, K. W., Kim, D., Park, H., & Jeong, B. (2017). Altered function of ventrolateral prefrontal cortex in adolescents with peer verbal abuse history. *Psychiatry investigation*, *14*(4), 441.

Lee, S. W., Yoo, J. H., Kim, K. W., Lee, J.-S., Kim, D., Park, H., Choi, J., & Jeong, B. (2015). Aberrant function of frontoamygdala circuits in adolescents with previous verbal abuse experiences. *Neuropsychologia*, *79*, 76-85.

Lim, L., Hart, H., Mehta, M. A., Simmons, A., Mirza, K., & Rubia, K. (2015). Neural correlates of error processing in young people with a history of severe childhood abuse: an fMRI study. *American Journal of Psychiatry*, *172*(9), 892-900.

Marusak, H. A., Etkin, A., & Thomason, M. E. (2015a). Disrupted insula-based neural circuit organization and conflict interference in trauma-exposed youth. *NeuroImage: Clinical*, *8*, 516-525.

Marusak, H. A., Martin, K. R., Etkin, A., & Thomason, M. E. (2015b). Childhood trauma exposure disrupts the automatic regulation of emotional processing. *Neuropsychopharmacology*, *40*(5), 1250-1258.

McCrory, E. J., De Brito, S. A., Kelly, P. A., Bird, G., Sebastian, C. L., Mechelli, A., Samuel, S., & Viding, E. (2013). Amygdala activation in maltreated children during pre-attentive emotional processing. *The British journal of psychiatry*, *202*(4), 269-276.

McLaughlin, K. A., Peverill, M., Gold, A. L., Alves, S., & Sheridan, M. A. (2015). Child maltreatment and neural systems underlying emotion regulation. *Journal of the American Academy of Child & Adolescent Psychiatry*, *54*(9), 753-762.

Miller, S., McTeague, L. M., Gyurak, A., Patenaude, B., Williams, L. M., Grieve, S. M., Korgaonkar, M. S., & Etkin, A. (2015). Cognition–Childhood maltreatment interactions in the prediction of antidepressant outcomes in major depressive disorder patients: Results from the iSPOT‐D trial. *Depression and anxiety*, *32*(8), 594-604.

Morelli, N. M., Liuzzi, M. T., Duong, J. B., Kryza-Lacombe, M., Chad-Friedman, E., Villodas, M. T., Dougherty, L. R., & Wiggins, J. L. (2021). Reward-related neural correlates of early life stress in school-aged children. *Developmental cognitive neuroscience*, *49*, 100963.

Mueller, S. C., Maheu, F. S., Dozier, M., Peloso, E., Mandell, D., Leibenluft, E., Pine, D. S., & Ernst, M. (2010). Early-life stress is associated with impairment in cognitive control in adolescence: an fMRI study. *Neuropsychologia*, *48*(10), 3037-3044.

Nagy, S. A., Kürtös, Z., Németh, N., Perlaki, G., Csernela, E., Lakner, F. E., Dóczi, T., Czéh, B., & Simon, M. (2021). Childhood maltreatment results in altered deactivation of reward processing circuits in depressed patients: a functional magnetic resonance imaging study of a facial emotion recognition task. *Neurobiology of stress*, *15*, 100399.

Neukel, C., Herpertz, S. C., Hinid-Attar, C., Zietlow, A.-L., Fuchs, A., Moehler, E., Bermpohl, F., & Bertsch, K. (2019). Neural processing of the own child’s facial emotions in mothers with a history of early life maltreatment. *European archives of psychiatry and clinical neuroscience*, *269*, 171-181.

Nicol, K., Pope, M., Romaniuk, L., & Hall, J. (2015). Childhood trauma, midbrain activation and psychotic symptoms in borderline personality disorder. *Translational Psychiatry*, *5*(5), e559-e559.

Park, A. T., Richardson, H., Tooley, U. A., McDermott, C. L., Boroshok, A. L., Ke, A., Leonard, J. A., Tisdall, M. D., Deater-Deckard, K., & Edgar, J. C. (2022). Early stressful experiences are associated with reduced neural responses to naturalistic emotional and social content in children. *Developmental cognitive neuroscience*, *57*, 101152.

Peters, A. T., Burkhouse, K. L., Kinney, K. L., & Phan, K. L. (2019). The roles of early-life adversity and rumination in neural response to emotional faces amongst anxious and depressed adults. *Psychological medicine*, *49*(13), 2267-2278.

Puetz, V. B., Viding, E., Gerin, M. I., Pingault, J.-B., Sethi, A., Knodt, A. R., Radtke, S. R., Brigidi, B. D., Hariri, A. R., & McCrory, E. (2020). Investigating patterns of neural response associated with childhood abuse v. childhood neglect. *Psychological medicine*, *50*(8), 1398-1407.

Puetz, V. B., Viding, E., Palmer, A., Kelly, P. A., Lickley, R., Koutoufa, I., Sebastian, C. L., & McCrory, E. J. (2016). Altered neural response to rejection‐related words in children exposed to maltreatment. *Journal of child psychology and psychiatry*, *57*(10), 1165-1173.

Quidé, Y., O’Reilly, N., Rowland, J. E., Carr, V. J., Elzinga, B. M., & Green, M. J. (2017). Effects of childhood trauma on working memory in affective and non-affective psychotic disorders. *Brain imaging and behavior*, *11*(3), 722-735.

Romens, S. E., Casement, M. D., McAloon, R., Keenan, K., Hipwell, A. E., Guyer, A. E., & Forbes, E. E. (2015). Adolescent girls’ neural response to reward mediates the relation between childhood financial disadvantage and depression. *Journal of child psychology and psychiatry*, *56*(11), 1177-1184.

Seghete, K. L. M., Kaiser, R. H., DePrince, A. P., & Banich, M. T. (2017). General and emotion-specific alterations to cognitive control in women with a history of childhood abuse. *NeuroImage: Clinical*, *16*, 151-164.

Seitz, K. I., Ueltzhöffer, K., Rademacher, L., Paulus, F. M., Schmitz, M., Herpertz, S. C., & Bertsch, K. (2023). Your smile won’t affect me: Association between childhood maternal antipathy and adult neural reward function in a transdiagnostic sample. *Translational Psychiatry*, *13*(1), 70.

Suzuki, H., Luby, J. L., Botteron, K. N., Dietrich, R., McAvoy, M. P., & Barch, D. M. (2014). Early life stress and trauma and enhanced limbic activation to emotionally valenced faces in depressed and healthy children. *Journal of the American Academy of Child & Adolescent Psychiatry*, *53*(7), 800-813. e810.

Takiguchi, S., Fujisawa, T. X., Mizushima, S., Saito, D. N., Okamoto, Y., Shimada, K., Koizumi, M., Kumazaki, H., Jung, M., & Kosaka, H. (2015). Ventral striatum dysfunction in children and adolescents with reactive attachment disorder: functional MRI study. *BJPsych open*, *1*(2), 121.

Taylor, S. E., Eisenberger, N. I., Saxbe, D., Lehman, B. J., & Lieberman, M. D. (2006). Neural responses to emotional stimuli are associated with childhood family stress. *Biological psychiatry*, *60*(3), 296-301.

Thomaes, K., Dorrepaal, E., Draijer, N., De Ruiter, M., Elzinga, B., Van Balkom, A., Smit, J., & Veltman, D. (2012). Treatment effects on insular and anterior cingulate cortex activation during classic and emotional Stroop interference in child abuse-related complex post-traumatic stress disorder. *Psychological medicine*, *42*(11), 2337-2349.

van Rooij, S. J., Smith, R. D., Stenson, A. F., Ely, T. D., Yang, X., Tottenham, N., Stevens, J. S., & Jovanovic, T. (2020). Increased activation of the fear neurocircuitry in children exposed to violence. *Depression and anxiety*, *37*(4), 303-312.

Wainsztein, A. E., Castro, M. N., Goldberg, X., Camacho-Téllez, V., Vulcano, M., Abulafia, C., Ladrón-de-Guevara, S., Cardoner, N., Nemeroff, C. B., & Menchón, J. M. (2021). Childhood adversity modulation of central autonomic network components during cognitive regulation of emotion in major depressive disorder and borderline personality disorder. *Psychiatry Research: Neuroimaging*, *318*, 111394.

Weissman, D. G., Jenness, J. L., Colich, N. L., Miller, A. B., Sambrook, K. A., Sheridan, M. A., & McLaughlin, K. A. (2020). Altered neural processing of threat-related information in children and adolescents exposed to violence: A transdiagnostic mechanism contributing to the emergence of psychopathology. *Journal of the American Academy of Child & Adolescent Psychiatry*, *59*(11), 1274-1284.

Weissman, D. G., Rosen, M. L., Colich, N. L., Sambrook, K. A., Lengua, L. J., Sheridan, M. A., & McLaughlin, K. A. (2022). Exposure to violence as an environmental pathway linking low socioeconomic status with altered neural processing of threat and adolescent psychopathology. *Journal of cognitive neuroscience*, *34*(10), 1892-1905.

Wymbs, N. F., Orr, C., Albaugh, M. D., Althoff, R. R., O’Loughlin, K., Holbrook, H., Garavan, H., Montalvo-Ortiz, J. L., Mostofsky, S., & Hudziak, J. (2020). Social supports moderate the effects of child adversity on neural correlates of threat processing. *Child abuse & neglect*, *102*, 104413.

Yang, R., Yu, Q., Owen, C. E., Aspe, G. I., & Wiggins, J. L. (2021). Contributions of childhood abuse and neglect to reward neural substrates in adolescence. *NeuroImage: Clinical*, *32*, 102832.
